# Urinary Inorganic Arsenic Concentration and Gestational Diabetes Mellitus in Pregnant Women from Arica, Chile

**DOI:** 10.3390/ijerph15071418

**Published:** 2018-07-05

**Authors:** María Pía Muñoz, Macarena Valdés, María Teresa Muñoz-Quezada, Boris Lucero, Paola Rubilar, Paulina Pino, Verónica Iglesias

**Affiliations:** 1 Escuela de Salud Pública, Facultad de Medicina, Universidad de Chile, 8380453 Santiago, Chile; mariapia.7@hotmail.com (M.P.M.); macavaldes@ug.uchile.cl (M.V.); paolarubilarr@gmail.com (P.R.); ppino@med.uchile.cl (P.P.); 2 Facultad de Ciencias de la Salud, Universidad Católica del Maule, 3480112 Talca, Chile; mtmunoz@ucm.cl (M.T.M.-Q.); balucero@gmail.com (B.L.)

**Keywords:** environmental exposure, inorganic arsenic, gestational diabetes, pregnant

## Abstract

*Introduction:* The association of total arsenic exposure with impaired glucose tolerance and gestational diabetes has been shown; however, evidence regarding urinary inorganic arsenic in pregnant women is still limited. Our aim was to evaluate the association between urinary inorganic arsenic concentration and gestational diabetes among pregnant women living in Arica, Chile. *Methods:* Cross-sectional study of pregnant women receiving care at primary health centers in urban Arica. The exposure was urinary inorganic arsenic concentration, while gestational diabetes was the outcome. The association was evaluated using multiple logistic regression models adjusted by age, education level, ethnicity, and pre-pregnancy body mass index. *Results:* 244 pregnant women were surveyed. The median urinary inorganic arsenic was 14.95 μg/L, and the prevalence of gestational diabetes was 8.6%. After adjusting, we did not find a significant association between gestational diabetes and inorganic arsenic exposure tertiles (Odds ratio (OR) 2.98, 95% CI = 0.87–10.18), (OR 1.07, 95% CI = 0.26–4.33). *Conclusion:* This study did not provide evidence on the relationship between urinary inorganic arsenic concentration and gestational diabetes. Further research is needed to elucidate the factors underlying this association.

## 1. Introduction

One of the pathologies of pregnancy that leads to a higher frequency of complications is diabetes mellitus (DM), a metabolic disease that erupts with increased blood glucose [1]. Gestational diabetes mellitus (GDM) occurs when the detection of diabetes is first achieved during gestation [2]. The increase of maternal diabetes prevalence has been in line with the increase of obesity and type 2 diabetes mellitus (DM2) [3,4,5]. A gestational diabetes prevalence of 7.6% has been observed in the United States [6]. In Chile, the prevalence of gestational diabetes has been estimated in 6.6% of pregnant women [7].

The magnitude of this condition is worrying because diabetes is associated with maternal complications during pregnancy and neonatal adverse outcomes, including pre-eclampsia, early delivery, congenital malformation, increased risk of intrauterine fetal death, fetal macrosomia, and neonatal hypoglycemia [8,9,10,11]. Women with GDM are more susceptible to developing type 2 diabetes mellitus after pregnancy [12], and children of women with hyperglycemia during pregnancy are at greater risk for developing obesity, glucose intolerance, and diabetes [13].

The most studied risk factors associated with non-insulin dependent diabetes mellitus are genetics, obesity, and lifestyle [1]. However, environmental pollutants have also been shown to play a role in this disease [14]. It is well-known that populations chronically exposed to high concentrations of arsenic through drinking water are at higher risk for developing diabetes mellitus [15,16,17], but controversy remains regarding moderate or low levels [18,19]. Studies have described the diabetogenic mechanism of arsenic, demonstrating its effect on pancreatic beta cell function and insulin sensitivity through oxidative stress, gene expression alteration, and inhibition of insulin signals involved in the uptake and transport of glucose. In addition, trivalent arsenic and its methylated metabolites (MMAIII and DMAIII) have been identified as the main harmful agents [20,21,22].

Some studies have reported an association between GDM and arsenic levels in maternal blood [23,24,25] or meconium [26]. However, information regarding gestational diabetes and urine inorganic arsenic is scarce. Ettinger et al. reported a non-significant association between arsenic in hair and glucose intolerance [23]. Farzan et al. evaluated the association between arsenic in water and arsenic in nails with the risk of impaired glucose tolerance and gestational diabetes, finding a significant association between nail arsenic and GDM. The authors stratified by body mass index (BMI) and reported a higher association between arsenic in water, GDM, and glucose intolerance in obese women [27]. Meanwhile, a French study showed that the odds of developing GDM was higher in pregnant women exposed to water arsenic ≥10 μg/L and, stratifying by pre-pregnancy BMI, also found a positive association in women with BMI ≥ 25 kg/m^2^ [28].

Water supply in Arica city in northern Chile, currently contains around 10 μg/L of arsenic [29]. However, since the late 1980s and for much of the 1990s, the city was also affected by pollution due to 21,000 tons of mineral residues with a high content of arsenic, lead, and cadmium that were left in the open, totally unprotected [30]. Although this toxic waste was eventually removed, suspicion remains that the soil could still be an additional source of exposure for its high arsenic content. The evaluation of other metals, such as lead, was not considered based on previous studies, which showed low exposure (range 1–9 μg/dL, median 2 μg/dL), unlike arsenic, which showed greater variability (range 2–78 μg/L, median 17.5 μg/L) [31].

The present study aims to evaluate the association between low to moderate inorganic arsenic exposure and gestational diabetes among women living in Arica, Chile.

## 2. Methods

### 2.1. Study Location and Subjects

This is a cross-sectional study of pregnant women, nested in a larger study whose participants were recruited from a list of pregnant women receiving care at the five existing family health centers in Arica. Women in their second trimester of gestation between June and October 2013 were invited to participate in the study; those who agreed signed an informed consent. The study was approved by the Ethics Committee of the Faculty of Medicine of the University of Chile and the Ethics and Scientific Committee of the Northern Health Zone, Chile.

### 2.2. Measurements

#### 2.2.1. Medical Record Data

Routine laboratory results recorded in the women’s medical charts were used to measure the outcome (i.e., gestational diabetes). A gestational diabetes diagnosis was given if fasting glycemia, assessed during the first prenatal visits was between 100 and 125 mg/dL on two different days and/or the oral glucose tolerance test (75 g of glucose) at 24–28 weeks of gestation was ≥140 mg/dL at 2 h. These criteria were established in the Pregnancy and Diabetes Guide by the Ministry of Health of Chile according to the diagnostic criteria of the World Health Organization (WHO) for diabetes [32,33]. The pre-pregnancy weight and height were also taken from their medical records to compute body mass index (BMI).

#### 2.2.2. Arsenic Exposure Assessment and Data Collection

The first morning spot urine sample was solicited to measure the urinary inorganic arsenic concentration. The participants were given verbal and written instructions for providing the sample. Once retrieved, the samples were stored in hermetically-sealed vials labeled with the participant’s identification (ID) and frozen at −20 °C until analysis at the University of Columbia laboratory. High-performance liquid chromatography with inductively coupled plasma mass spectrometry (HPLC–ICPMS) was used to measure the concentration of arsenite (AsIII), arsenate (AsV), and their methylated metabolites monomethylarsonic (MMA) and dimethylarsinic (DMA) acids. The limit of detection (LOD) was 0.1 μg/L for each metabolite. Concentrations below the LOD were assigned a value of half of the LOD (0.05 μg/L). Total urinary inorganic arsenic concentration (T-InAs) was calculated by adding the values of the species AsIII (μg/L), AsV (μg/L), MMA (μg/L), and DMA (μg/L). The inorganic arsenic (InAs: AsIII + AsV), MMA, and DMA were divided respectively by total urinary inorganic arsenic to obtain the percentages. Also, methylation indices were calculated; primary methylation index (PMI) was defined as the ratio of MMA and InAs, and secondary methylation index (SMI) was defined as the ratio between DMA and MMA.

#### 2.2.3. Maternal Questionnaire

A questionnaire was applied to gather the sociodemographic information (age, years of formal education, ethnicity, family income, and marital status), environmental exposure (sources of drinking and cooking water, residential history, and fish consumption in the last 72 h). Tobacco and alcohol consumption were also inquired into as part of the larger study.

### 2.3. Statistical Analysis

After thorough exploratory and descriptive analysis, the association between urinary inorganic arsenic concentration and gestational diabetes was appraised through multiple logistic regression models adjusting for age, ethnicity, education level, and pre-pregnancy BMI. These covariates were considered as potential confounders based on existing grounded literature [23,24,26]. We conducted further analyses evaluating whether the BMI performs as an effect modifier of arsenic on gestational diabetes. The results are presented as odds ratios (OR) and respective 95% confidence intervals (CI). Data were analyzed using the statistical package STATA v12.0 (StataCorp., College Station, TX, USA).

## 3. Results

Of the 257 recruited pregnant women, 246 had complete diabetes testing in their medical record, but two were excluded because they had pre-diabetes. There were no differences in any important features when comparing the group with complete and incomplete diabetes data. The characteristics of the pregnant women by diabetes status are described in Table 1.

The prevalence of gestational diabetes was 8.6% (*n* = 21). As shown in Table 1, there were no significant differences regarding individual traits among women with and without GDM (Table 1).

Urine samples were obtained from 230 participants. The median for total urinary arsenic concentration was 14.95 μg/L (interquartile range 10.6–23.1 μg/L). Table 2 presents the median and interquartile range for the total urinary inorganic arsenic concentration (T-InAs), the mean percentages of InAs, MMA, and DMA, and the methylation indices by GMD status. As expected, dimethylarsinic acid (DMA) largely represents most of the total inorganic arsenic. No differences were observed in the urinary arsenic species percentages and the methylation indices among both groups.

The total urinary inorganic arsenic concentration values did not show a normal distribution (*p* ≤ 0.001, Shapiro-Wilk test) with minimum–maximum values of 2.05 and 69.3 μg/L. For that reason, these values were categorized in tertiles as follows: T1 (lower), 2.05–11.08 μg/L, (*n* = 77); T2 (middle), 11.09–19.90 μg/L (*n* = 77); and T3 (upper), 19.91–69.30 μg/L (*n* = 76). The prevalence of gestational diabetes was 5.2% in the lowest tertile, 14.3% in the intermediate, and 6.6% in highest.

No association was found when we modeled urinary inorganic arsenic as a continuous variable (OR = 0.98; 95% IC = 0.94–1.03), nor after logarithmic transformation. In terms of tertile of exposure, the crude analysis shows that total inorganic arsenic exposure at tertile levels 2 and 3 increases the risk of gestational diabetes (OR = 3.04; 95% CI = 0.92–10.02 and OR = 1.28, 95% CI = 0.33–4.98, respectively); however, the relation was not statistically significant. After adjusting for age, education, ethnicity, and BMI the results did not change considerably (Table 3).

We also considered the variables “type of drinking water” and “fish consumption in the last 72 h” aiming at eliminating the contribution of organic arsenicals from recent fish intake to DMA concentration, which rose to 6.47 (95% CI = 1.27–32.73), while for T3 the OR remained not significant (95% CI = 0.15–9.34). Notwithstanding, this analysis is hampered by the loss of an important fraction of the sample, because we only have the information on the food intake of 182 participants, restricting the original 230 pregnant women to just 169.

Finally, we evaluated whether the BMI was a variable that modifies the association between total urinary arsenic concentration and gestational diabetes in pregnant woman, but the interaction term was not significant.

## 4. Discussion

The results suggest that moderate exposure to inorganic arsenic prevails among the participants, with a median of total urinary inorganic arsenic concentration of 14.95 μg/L, while the prevalence of gestational diabetes was 8.6%. Estimates of gestational diabetes in Chile are scarce; a recent study reported a prevalence of 6.6% [7], lower than in the current study. Our research did not show an association between gestational diabetes and total urinary inorganic arsenic levels. When we assessed T-InAs in tertiles, we found a higher risk in the second and third tertile of exposure, but both were not significant. When we modeled urine inorganic arsenic as a continuous variable the association was not significant either.

The results of studies evaluating the association between exposure to arsenic and diabetes mellitus have been consistent when levels of arsenic in water exceed 500 μg/L [15,16], but with moderate levels of exposure, the results are not clear. While some studies have shown that moderate arsenic exposure is a risk factor of diabetes [18,34,35], others have not found an association, or their results are inconsistent [19,36,37].

Investigations that have evaluated the association between inorganic arsenic exposure and its relationship with GDM are scarce [23,27,28]. The present study is one of the few that provides evidence with total urine inorganic arsenic concentration as the exposure variable. Indeed, previous studies showing significant associations have used arsenic in meconium (total arsenic) [26], nail (inorganic arsenic) [27], water (inorganic arsenic) [27,28], and blood (total arsenic) [23,24,25] as the exposure variable. The only study that reported inorganic arsenic in urine as the exposure variable did not find a consistent and significant association [27].

As we described above, contrasting with other studies in which the strongest association between gestational diabetes and arsenic was for the highest exposure category [23,24,25,26], in our study we did not find a dose–response association. Ettinger et al. reported an association between glucose intolerance and the quartile of highest arsenic blood exposure (OR = 2.8; 95% CI = 1.1–6.9) [23], and Shapiro et al. also found an association between gestational diabetes and the quartile of highest exposure to blood arsenic concentration (OR = 3.7; 95% CI = 1.4–9.6) [24]. Xia et al., in a recent cohort study, revealed an association between blood arsenic concentration and GDM in the 4th quartile (OR = 1.7; 95% CI 1.23–2.38) [25]. Besides relating arsenic in blood to glucose intolerance, Ettinger et al. studied this outcome’s association with arsenic in hair. They found a significant association with quartiles 2 and 3 but not with the highest (OR = 4.20; 95% CI = 0.74–23.86) [23]. In a cohort study of pregnant women in New Hampshire, Farzan et al. evaluated the association between inorganic arsenic as a continuous variable and the risk of glucose intolerance and gestational diabetes. They found a significant association between arsenic concentration in nails and gestational diabetes (OR = 4.5; 95% CI = 1.2–16.6). Additionally, they reported a positive association between water arsenic concentration and gestational diabetes (OR = 1.1; 95% CI = 1.0–1.2) when a stratified analysis by BMI was added between arsenic in water, GDM, and glucose intolerance, evidencing a greater association in obese women (OR = 1.7; 95% CI = 1.0–2.8). They also evaluated the relationship with urine inorganic arsenic concentration (as a continuous variable) without observing an association [27], as in our study.

It is interesting to note in this last study that with an average of 4.2 μg/L of arsenic in water (range: 0.001–189.3 μg/L) and an average of 5.9 μg/L in urine (range 0.2–288.5 μg/L), the authors found a significant association of GDM and arsenic in water but not with arsenic in urine, although both indicators were correlated [27]. This result makes us reflect on the pertinence of using arsenic in urine as a biomarker of exposure compared to the use of arsenic in water or nail, which could better represent a cumulative exposure. Indeed, in a study recently published in which the authors evaluated the association between GDM and arsenic in water, only a 7.3% of the population were exposed to levels over 10 μg/L, and a positive association was reported [28]. In our study, the aim was to evaluate the association between total urinary inorganic arsenic concentration and gestational diabetes in an urban commune where the level of arsenic in the water meets the standard of 10 μg/L [38]. That is why just a small number of water samples (*n* = 5) were measured in an exploratory manner, which prevent us from doing a further analysis to evaluate *a* posteriori this association. However, when the analysis was done incorporating the variable of “how many servings of tap water consume per day” (as a continuous variable) the results show a significant association, even after adjusting by the same variables described in the model in the Table 3 (OR_adj_ = 1.15; 95% CI = 1.01–1.29). This result should be viewed with caution, because one of the symptoms of diabetes is thirst, so it is likely that we could be in presence of a reverse causality bias.

Regarding the profile analysis of urinary concentrations of inorganic arsenic metabolites, DMA was close to 83%. This percentage was consistent with results from other studies that measured the urinary profile of inorganic arsenic metabolites during pregnancy [39,40,41]. We further analyze the association with each metabolite, the percentage of MMA and DMA, and with the primary and secondary methylation index. None was significantly associated with the risk of gestational diabetes (data not shown). As informed, we adjusted for the consumption of fish, given the changeover of organic to inorganic DMA of a fraction of arsenic from seafood reported by Navas-Acien et al. [42]. That analysis increased significantly the association with T2 arsenic exposure, but we remain cautious on the importance of this finding, because it is based on just 70% of the original sample.

A potential limitation is the lack of urinary creatinine concentration. This variable is sometimes used to adjust urinary inorganic arsenic values for variations in hydration or other factors that may influence concentration. However, it has been shown that adjusting for creatinine did not affect the correlation between total urinary inorganic arsenic concentration and arsenic levels in the environment in first morning samples or 24-h samples of urine [43]. Moreover, creatinine excretion has been found to be abnormal in patients with DM, and therefore, this method could distort the association between inorganic arsenic concentration and diabetes [19,44]. In relation to a potential selection bias, although there was no random selection of participants (all pregnant women from the list provided by health centers were invited to participate) there is no reason to assume a differentiated participation between those who developed or not diabetes during gestation. Regarding the measurement of the exposure variable, it would have been interesting to have a systematic measurement of the concentration of arsenic in the water. As mentioned previously, the measurement of this variable was not considered in the larger study, rather it was measured in an exploratory manner and not concurrently with the urine sample, which did not allow us to evaluate its relationship with gestational diabetes as done in other studies.

The small sample size available to evaluate the association between inorganic arsenic and gestational diabetes resulted in wide confidence intervals. These results suggest that the number of pregnant women studied was insufficient to demonstrate an association, affecting the power of the study. Indeed, if we consider the small number of cases and that the proportion of exposed in the group of GDM and without GDM is similar, the power to demonstrate association is around 20%. Although it was possible to identify a greater risk (not significant) in the second tertile of exposure, the question remains whether the dose response relationship would have been shown if the sample size was larger.

The strengths of our study include the fact that this is one of the first to evaluate the association between urine inorganic arsenic concentration at low-middle levels and gestational diabetes. Furthermore, the speciation of arsenic concentration allowed us to quantify the inorganic component of the total arsenic. The use of tests recorded in the women’s medical charts support the validity of the outcome variable. We also had variables like pre-pregnancy weight and height which allowed us to calculate body mass index (BMI), included in the analysis as a confounding variable.

## 5. Conclusions

This study did not provide evidence of an association between total urinary inorganic arsenic concentration and gestational diabetes. Because inorganic arsenic levels during pregnancy represents an increased risk both in the development of other long-term diseases in woman and in early exposure of the unborn child, it is important to investigate this pollutant as an additional risk factor. Further research is needed to elucidate the factors underlying the association between inorganic arsenic exposure and gestational diabetes.

## Figures and Tables

**Table 1:** Characteristics of pregnant women according to gestational diabetes mellitus (GDM) status, Arica 2013–2014.

Caption	Non-GDM, (*n* = 223)	GDM, (*n* = 21)	*p*-Value *
	*n* (%)	*n* (%)	
Age (year)			
≤29 years	166 (74.4)	12 (57.1)	
30–34 years	35 (15.7)	6 (28.6)	
≥35 years	22 (9.9)	3 (14.3)	0.17
Education			
≤12 years	136 (5.4)	17 (81.0)	
>12 years	87 (94.6)	4 (19.0)	0.07
Ethnicity			
White	144 (64.6)	15 (71.4)	
Aymara	65 (29.1)	5 (23.8)	
Other	14 (6.3)	1 (4.8)	0.93
Marital status			
Married/living with partner	97 (43.5)	10 (47.6)	
Single/separated/divorced	126 (56.5)	11 (52.4)	0.71
Pre-pregnancy BMI (Kg/m^2^)			
Normal (≤24.9)	127 (57.0)	7 (33.3)	
Overweight (25.0–29.9)	60 (26.9)	8 (38.1)	
Obese (>30.0)	36 (16.1)	6 (28.6)	0.08
Parity			
Nulliparous	104 (46.6)	9 (42.9)	
1 and 2	104 (46.6)	9 (42.8)	
≥3	15 (6.8)	3 (14.2)	0.38
Smoked during pregnancy			
Smoked	14 (6.3)	2 (9.5)	0.22
Type of drinking water			
Tap water	80 (35.8)	10 (47.6)	0.28
Fish intake in the last 72 h	23 (14.5)	2 (14.3)	0.97

* Pearson Chi-square test and Fisher’s exact test for categorical variables. BMI = body mass index.

**Table 2:** Comparison of total urinary inorganic arsenic concentration, the percentages of the arsenic species and methylation indices, Arica 2013–2014.

Caption	Non-GDM (*n* = 223)	GDM (*n* = 21)	*p*-Value *
	Median (P_25_–P_75_)	Median (P_25_–P_75_)	
T-InAs (μg/L)	15.12 (10.5–23.4)	14.72 (12.8–20.4)	0.96
	**Mean ± SE**	**Mean ± SE**	
InAs%	8.5 ± 0.4	7.5 ± 0.9	0.55
MMA%	8.6 ± 3.9	8.9 ± 3.5	0.59
DMA%	82.8 ± 0.5	83.6 ± 1.5	0.78
PMI	1.8 ± 0.2	1.7 ± 0.3	0.20
SMI	14.6 ± 2.4	12.0 ± 1.7	0.69

* Wilcoxon rank-sum (Mann-Whitney) test for continuous variables. InAs = inorganic arsenic; MMA = methylated metabolites monomethylarsonic acid; DMA = dimethylarsinic acid; PMI = primary methylation index; and SMI = secondary methylation index.

**Table 3:** Crude and adjusted association between the concentration of total inorganic arsenic in urine and gestational diabetes, Arica 2013–2014.

Caption	*n*	% GDM	Crude OR (95% CI)	Adjusted OR (95% CI) ^a^
T-InAs (μg/L)				
T1 (2.05–11.08)	77	5.2	1.00	1.00
T2 (11.09–19.90)	77	14.3	3.04 (0.92–10.02)	2.98 (0.87–10.18)
T3 (19.91–69.30)	76	6.6	1.28 (0.33–4.98)	1.07 (0.26–4.33)

^a^ Adjusted for age (1: <30 years, 2: 30–34 years, and 3: ≥35 years), education (0: >12 years, 1: ≤12 years), ethnicity (1: white, 2: aymara/other), and pre-pregnancy BMI (kg/mt^2^). OR = odds ratio.
